# Molecular Detection and Characterization of *Rickettsia asembonensis* in Human Blood, Zambia

**DOI:** 10.3201/eid2708.203467

**Published:** 2021-08

**Authors:** Lavel C. Moonga, Kyoko Hayashida, Namwiinga R. Mulunda, Yukiko Nakamura, James Chipeta, Hawela B. Moonga, Boniface Namangala, Chihiro Sugimoto, Zephaniah Mtonga, Mable Mutengo, Junya Yamagishi

**Affiliations:** Hokkaido University, Sapporo, Japan (L.C. Moonga, K. Hayashida, Y. Nakamura, C. Sugimoto, J. Yamagishi);; University of Zambia, Lusaka, Zambia (N.R. Mulunda, J. Chipeta, B. Namangala, M. Mutengo);; National Malaria Control Center, Lusaka (H.B. Moonga);; Chongwe District Hospital, Lusaka (Z. Mtonga);; Levy Mwanawasa Medical University, Lusaka (M. Mutengo)

**Keywords:** *Rickettsia asembonensis*, fleaborne rickettsiosis, *Rickettsia felis*-like organisms, Rickettsemia, multiple-gene sequencing, Zambia, vector-borne diseases, rickettsial diseases, bloodborne pathogens

## Abstract

*Rickettsia asembonensis* is a flea-related *Rickettsia* with unknown pathogenicity to humans. We detected *R. asembonensis* DNA in 2 of 1,153 human blood samples in Zambia. Our findings suggest the possibility of *R. asembonensis* infection in humans despite its unknown pathogenicity.

*Rickettsia asembonensis* is a fleaborne rickettsia closely related to *Rickettsia felis* and is thus referred to as an *R. felis*–like organism. *R. asembonensis* was first detected in cat fleas in Kenya and subsequently reported worldwide (*1*,*2*). Although *R. felis* has been increasingly recognized as a human infective agent that can cause human febrile disease, the infectivity and pathogenicity of *R. asembonensis* in humans is largely unknown. Recent investigations in patients with febrile illness and petechial lesions identified *R. asembonensis* DNA and antibodies for rickettsial antigens in Malaysia (*3*,*4*). Furthermore, *R. asembonensis* was isolated in cellular cultures from patients in Peru with acute febrile illness and confirmed by sequencing (*5*). These reports suggest the possibility of *R. asembonensis* as a human infective agent. However, no direct evidence of *R. felis* and *R. asembonensis* as an etiologic agent of human illness has been established. A previous study in Zambia revealed the predominant existence of *R. asembonensis* and *R. felis* in cat fleas (*6*). Our study investigates the presence of these rickettsiae in human blood in Zambia.

We obtained 753 residual patient blood samples from hospitals in urban Lusaka (n = 519) and the Chongwe District (n = 234) of Zambia. Approximately half of the samples (303/753) were traceable to clinical records of patients. The common clinical conditions among these patients included fever, anemia, meningitis, septicemia, and sickle cell anemia (Appendix Table 1). In addition, we obtained dried blood spots on Whatman FTA classic cards (Millipore Sigma, https://www.sigmaaldrich.com) from healthy volunteers from rural eastern (n = 200) and central (n = 200) provinces to assess rickettsia infection in healthy rural persons. The study was approved by the National Health Research Authority of Zambia through the Biomedical Research Ethics Committee (reference no. 007-10-18).

We extracted genomic DNA and subjected it to PCR screening that targeted the citrate synthase gene (*gltA*) of *Rickettsia*. We subjected the positive samples to multiple-gene sequencing analysis targeting the 17-kDa common antigen (*htrA*), outer membrane protein A (*OmpA*), and outer membrane protein B (*OmpB*) genes using previously described primers (Appendix Table 2). We aligned the sequences using MAFFT (https://mafft.cbrc.jp/alignment/server) and performed phylogenetic analysis by the neighbor-joining method using MEGA7 (https://www.megasoftware.net). We determined the estimated *Rickettsia* bacterial burden in *Rickettsia-*positive blood samples by *OmpA* quantitative PCR by using published primers. We further testsed the *gltA* PCR-positive samples for malaria by nested PCR (Appendix Table 2).

We detected *R. asembonensis* in 0.39% (2/519) samples from the urban Lusaka District by *gltA* PCR. The samples from the Chongwe District and the rural areas of the eastern and central provinces were all negative, although the possibility that dried blood spot samples from rural areas might have lower detection sensitivity cannot be ruled out. BLAST analysis (https://blast.ncbi.nlm.nih.gov/Blast.cgi) of the *gltA* sequences obtained (GenBank accession nos. LC557154 and LC557155) showed 100% homology to *R. asembonensis* identified in cat fleas from human dwellings and domestic dogs in 3 countries: Senegal (GenBank accession no. JF966774), Kenya (accession no. JN315968), and Zambia (accession no. LC431490) (*6*,*7*). Comparing the sequenced *gltA* with those detected in fleas from Peru (GenBank accession no. KY650697) and other regions in the Americas showed 99.8% similarity. Phylogenetic analysis of *gltA* confirmed the detected sequences’ closer relatedness by clustering with genes from cat fleas from sub-Saharan Africa, a distinct cluster from other regions (Figure). The *OmpA*, *OmpB*, and *htrA* sequences showed clustering without regional discrimination (data not shown). The obtained nucleotides are available in GenBank under accession nos. LC557154–61. Detection of genotypically similar *R. asembonensis* in persons and cat fleas in Zambia suggests possible human infection by *R. asembonensis* through cat flea bites. Nevertheless, the epidemiologic cycle and pathogenicity of *R. asembonensis* and other related *R. felis*–like organisms remain to be elucidated.

**Figure:**
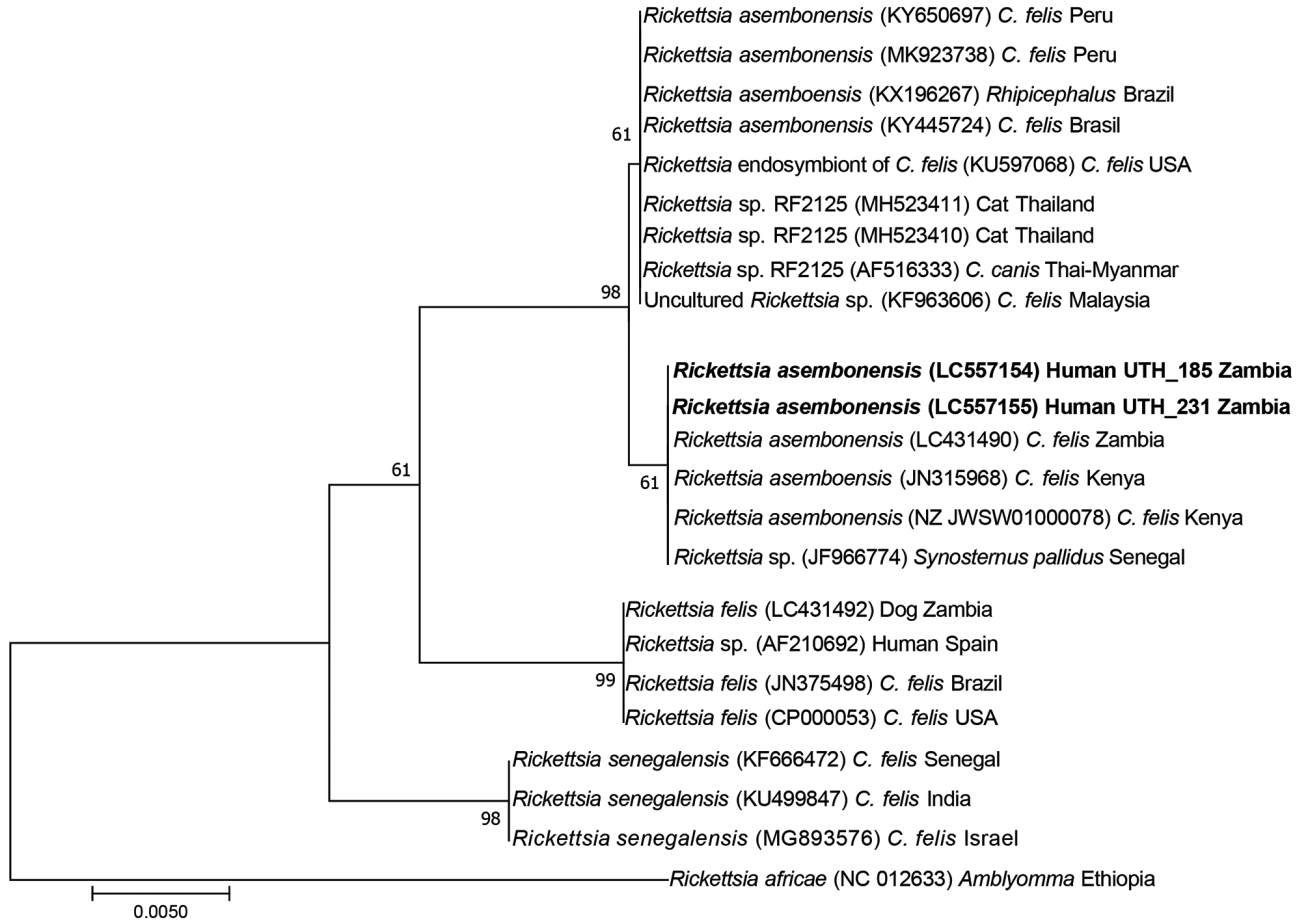
Phylogenetic tree of *Rickettsia felis* and *R. felis–*like organisms based on the sequences of the *gltA* gene (581 bp) from human blood samples collected from Zambia, 2019 (in bold). The tree was constructed using the neighbor-joining method with the maximum-likelihood model. Bootstrap values are shown on nodes based on 1,000 replicates. Sequences are identified by species name, GenBank accession number, host, and country of detection. Scale bar indicates nucleotide substitutions per site.

The patient identified as UTH_185 in whom *R. asembonensis* was detected had a medical record of anemia and weight loss (Table). The malaria test was negative. Despite the limited association of *Rickettsia* infection with anemia, severe *R. felis* infection has been reported with severe anemia, possibly attributable to hemorrhage from vascular damage in rickettsial disease (*8*). However, the observed evidence was limited and could not establish *R. asembonensis* as the cause of these symptoms. The second *R. asembonensis*–positive sample from the patient identified as UTH_231 had limited clinical information, which did not allow for further interpretation. The 2 *R. asembonensis*–positive blood samples showed estimated DNA quantities of 890,000 copies/mL of blood from patient UTH_185 and 2,100,000 copies/mL of blood from patient UTH_231 (Table). These results are within the same range as a previous study for *Rickettsia rickettsii* estimated rickettsial burden (*9*).

**Table:** Selected demographic and clinical characteristics of 2 persons in whom *Rickettsia asembonensis* was detected from blood samples collected in Zambia

Characteristic	Patient** UTH_185**	Patient** UTH_231**
Age, y	42	45
Sex	Female	Female
Residential area	Lusaka	Lusaka
Clinical manifestation	Anemia and weight loss	No information
Estimated rickettsia genome copies/mL blood	890,000	2,150,000
Malaria test	Negative	Negative

In conclusion, detection of *R. asembonensis* of identical genotype in cat fleas and human blood in Zambia suggests possible transmission from cat fleas to humans. Given the worldwide distribution of *R. asembonensis*, further studies to elucidate its pathogenicity and epidemiologic cycle are warranted.

Appendix Information related to the molecular detection and characterization of *Rickettsia asembonensis* in human blood collected in Zambia, 2019.
