# Supplementary material for: Molecular Detection and Characterization of Rickettsia asembonensis in Human Blood, Zambia
Source: Emerg Infect Dis. 2021 Aug;27(8):2237–9. doi: 10.3201/eid2708.203467 (PMC8314825; doi:10.3201/eid2708.203467)
Supplement: Appendix — Information related to the molecular detection and characterization of Rickettsia asembonensis in human blood collected in Zambia, 2019. [file 20-3467-Techapp-s1.pdf]

Article DOI: https://doi.org/10.3201/eid2708.203467

# Molecular Detection and Characterization of *Rickettsia asembonensis* Human Blood, Zambia

## Appendix

**Appendix Table 1.** Classification of clinical manifestation of sampled individuals obtained from hospitals in Lusaka and Chongwe, Zambia.

| Clinical manifestation or disease name | Number of samples in the category |
|---|---|
| Febrile illness | 10 |
| Neurologic symptoms (meningitis) | 12 |
| Septicemia | 22 |
| Anemia | 36 |
| Sickle cell anemia | 45 |
| HIV | 18 |
| Malaria | 24 |
| Respiratory infection | 49 |
| Others | 87 |
| No clinical information provided | 450 |
| **Total** | **753** |

**Appendix Table 2.** Primers for amplification of genes from *Rickettsia* spp. and *Plasmodium* spp

| Target gene | Target pathogen | Annealing temperature (°C) | Amplicon size (bp) | Oligonucleotide sequence (5′-3′) | Reference |
|---|---|---|---|---|---|
| Citrate synthase gene (*gltA*) | *Rickettsia* genus | 54 | 581 | Forward: gltA_Fc CGAACTTACCGCTATTAGAATG Reverse: gltA_Rc CTTTAAGAGCGATAGCTTCAAG | (*1*) |
| Outer membrane *A* gene (*ompA*)* | Spotted fever group rickettsia | 48 | 258 | Forward: ompA_Fm TGCAGGGCTTAGATATTCGGC Reverse: ompA_Rm AAGCTGTTGGTAAAGGAGCA | (*2*) |
| Outer membrane protein *B* gene (*ompB*) | Spotted fever group rickettsia | 48 | 776 | Forward: ompB_Fm GGACCTGAAGCTGGAGCAAT Reverse: ompB_Rm CTGTCAGGCTGGCTGATGAA | (*2*) |
| 17kDa common antigen gene (*htrA*) | *Rickettsia* genus | 50 | 545 | Forward: 17k_5 GCTTTACAAAATTCTAAAAACCATATA Reverse: 17k_3 TGTCTATCAATTCACAACTTGCC | (*3*) |
| Small subunit ribosomal RNA gene (SSU rRNA) | *Plasmodium* genus | 60 | 620 | Forward: rPLU 1 TCAAAGATTAAGCCATGCAAGTGA Reverse: rPLU 5 CCTGTTGTTGCCTTAAACTTC | (*4*) |
| Small subunit ribosomal RNA genes (SSU rRNA) | *Plasmodium* genus (nested-PCR) | 60 | 240 | Forward rPLU 3: TTTTTATAAGGATAACTACGGAAAAGCTGT Forward rPLU 4: TACCCGTCATAGCCATGTTAGGCCAATACC | (*4*) |

*OmpA* primer set used for estimation of rickettsial load by quantitative real-time PCR using SYBR Green assay.

**References**


1. Gaowa, Ohashi N, Aochi M, Wuritu D, Wu DX, Yoshikawa Y, et al. *Rickettsiae* in ticks, Japan, 2007–2011. Emerg Infect Dis. 2013;19:338–40. PubMed https://doi.org/10.3201/eid1902.120856

2. Moonga LC, Hayashida K, Nakao R, Lisulo M, Kaneko C, Nakamura I, et al. Molecular detection of *Rickettsia felis* in dogs, rodents and cat fleas in Zambia. Parasit Vectors. 2019;12:168. PubMed https://doi.org/10.1186/s13071-019-3435-6

3. Labruna MB, Whitworth T, Bouyer DH, McBride J, Camargo LM, Camargo EP, et al. *Rickettsia bellii* and *Rickettsia amblyommii* in *Amblyomma* ticks from the state of Rondônia, Western Amazon, Brazil. J Med Entomol. 2004;41:1073–81. PubMed https://doi.org/10.1603/0022-2585-41.6.1073

4. Singh B, Bobogare A, Cox-Singh J, Snounou G, Abdullah MS, Rahman HA. A genus- and species-specific nested polymerase chain reaction malaria detection assay for epidemiologic studies. Am J Trop Med Hyg. 1999;60:687–92. PubMed https://doi.org/10.4269/ajtmh.1999.60.687
